# Probiotics and Synbiotics Supplementation Reduce Inflammatory Cytokines in Individuals with Prediabetes and Type 2 Diabetes Mellitus: Findings from a Systematic Review Meta-analysis

**DOI:** 10.1016/j.advnut.2025.100526

**Published:** 2025-09-27

**Authors:** Azin Setayesh, Mehdi Karimi, Fereshteh Valizadeh, Omid Asbaghi, Samira Pirzad, Sayed Hossein Davoodi, Bagher Larijani

**Affiliations:** 1 Department of Community Nutrition, School of Nutritional Sciences and Dietetics, Tehran University of Medical Sciences, Tehran, Iran; 2 Faculty of Medicine, Bogomolets National Medical University, Kyiv, Ukraine; 3 Faculty of Medicine, Shahid Beheshti University of Medical Sciences, Tehran, Iran; 4 Cancer Research Center, Shahid Beheshti University of Medical Sciences, Tehran, Iran; 5 Faculty of Medicine, Islamic Azad University, Tehran Medical Sciences Branch, Tehran, Iran; 6 National Nutrition and Food Technology Research Institute, Shahid Beheshti University of Medical Sciences, Tehran, Iran; 7 Endocrinology and Metabolism Research Center, Endocrinology and Metabolism Clinical Sciences Institute, Tehran University of Medical Sciences, Tehran, Iran

**Keywords:** probiotic, synbiotic, diabetes, prediabetes, inflammation, cytokines, nutrition, meta-analysis

## Abstract

Chronic inflammation plays a significant role in the development and progression of diabetes. Despite growing interest in probiotic and synbiotic supplementation, there is limited consensus on their efficacy in modulating inflammatory cytokines. This meta-analysis evaluates the impact of these supplements on inflammatory cytokines in adults with prediabetes and type 2 diabetes mellitus (T2DM). A comprehensive search was conducted on online databases from their inception to September 2025 to identify relevant randomized controlled trials (RCTs). Data were extracted from selected studies, and the overall effect size was determined using weighted mean differences (WMD) with 95% confidence intervals (CIs) through a random-effects model. The pooled analysis of 22 RCTs, including 1321 individuals with prediabetes and T2DM, showed that probiotic and synbiotic supplementation significantly reduced C-reactive protein (CRP) (WMD: −0.46 mg/L, 95% CI: [−0.77, −0.15], p=0.003), interleukin-6 (IL-6) (WMD: −0.43 pg/ml, 95% CI: [−0.76, −0.09], p=0.012), and tumor necrosis factor-alpha (TNF-α) (WMD: −1.42 pg/ml, 95% CI: [−2.15, −0.69], p<0.001). Subgroup analyses revealed that CRP reduction was greatest among participants with baseline CRP ≥3 mg/L, those undergoing longer interventions (≥12 weeks), individuals with T2DM, overweight participants, and when probiotics were administered. IL-6 levels were significantly reduced in obese individuals, particularly with longer treatment durations and synbiotic interventions, while TNF-α reductions were most pronounced in long-term interventions (≥12 weeks), especially among T2DM patients with normal BMI and when probiotics were used. In conclusion, probiotic and synbiotic supplementation significantly reduces inflammatory cytokines (CRP, IL-6, TNF-α) in individuals with prediabetes and T2DM, with the strongest effects observed in those with higher baseline inflammation and longer intervention durations, underscoring the importance of tailoring supplementation strategies to individual inflammation status, intervention duration, and metabolic profile to optimize therapeutic outcomes.

**Statement of Significance**

This systematic review represents the most comprehensive and up-to-date meta-analysis evaluating the effects of probiotic and synbiotic supplementation on key inflammatory cytokines (C-reactive protein, IL-6, and TNF-α) in individuals with prediabetes and type 2 diabetes mellitus. Through the inclusion of dose–response analyses, subgroup analyses based on baseline inflammation, BMI, and intervention duration, as well as a Grading of Recommendations, Assessment, Development, and Evaluations assessment of evidence quality, this work provides a robust and clinically relevant synthesis to inform personalized supplementation strategies for optimal anti-inflammatory and metabolic outcomes.

## Introduction

Type 2 diabetes mellitus (T2DM) is a noncommunicable disease that has become an ever-growing public health concern, estimated to cross 700 million by 2045 [1]. Prediabetes is a preclinical state characterized by impaired glucose tolerance and impaired fasting glucose levels. According to statistics, ∼70% of patients with prediabetes eventually develop T2DM [2]. Environmental and genetic factors play an imperative role in the development of T2DM [3]. Also, the potential association of inflammation with diabetes susceptibility and its complications has been shown by developing evidence [4]. If the disease remains uncontrolled, it might cause comorbidities, including cardiovascular disease, retinopathy, neuropathy, and kidney failure [5].

Given the rising burden of T2DM, there is an urgent need for comprehensive management strategies that incorporate pharmaceutical interventions, nutritional supplementation, and lifestyle modifications. Successful management of T2DM requires an ongoing commitment, consistent medical monitoring, and strict adherence to prescribed treatment plans. In recent years, numerous studies have investigated the benefits of dietary supplements as adjuncts to conventional therapies, with many demonstrating promising results in improving glycemic control and overall disease outcomes [[6], [7], [8], [9], [10], [11]].

Emerging evidence has focused on the effects of intestinal microbiota composition and its metabolites on glycemic, metabolic, and inflammatory biomarkers. Therefore, diet-related interventions, such as probiotics, prebiotics, and synbiotics, are practical strategies to modulate the gut microbiota through various mechanisms [12,13]. Probiotics are live microorganisms, such as *Lactobacillus* and *Bifidobacteria*, added to many supplements [14,15]. Prebiotics are nondigestible food components, such as large polysaccharides, fructooligosaccharides, and lactulose, which selectively stimulate the growth and activation of probiotic bacteria [16]. The combination of prebiotics and probiotics is described as a synbiotic that acts synergistically on the host's metabolic health [17].

Probiotics and synbiotics play a promising role in modulating inflammation and altering gene expression in patients with T2DM by increasing microbial fermentation of fibers in the colon and producing short-chain fatty acids (SCFAs). SCFAs modulate gut permeability, regulate the expression of inflammatory cytokines, blood pressure [18], and affect glucose homeostasis [19,20]. Some studies support the beneficial effects of modulating glycemic status and pre- and proinflammatory cytokines [21,22]. However, the mechanism is not yet fully understood, and there are inconsistent views regarding the beneficial effects of probiotics and synbiotics.

Although previous studies have highlighted the positive effects of probiotic and synbiotic supplements on metabolic control in T2DM, limited and inconsistent evidence exists regarding their impact on inflammatory mediators. To address this gap, our study aims to evaluate the dose–response relationship between these supplements and the regulation of inflammatory biomarkers.

## Methods

### Study design and protocol

This study was performed according to the PRISMA guideline [23]. The study was designed based on the Population, Intervention, Comparison, Outcomes, Study framework [24], including:

- P (Population): adult individuals with prediabetes and T2DM
- I (Intervention): probiotics and synbiotics oral supplementation
- C (Comparison): placebo, no supplementation, or control
- O (Outcomes): changes in inflammatory cytokines, including C-reactive protein (CRP), interleukin-6 (IL-6), and tumor necrosis factor-alpha (TNF-α)
- S (Study design): randomized controlled trials (RCTs).

### Search strategy

A comprehensive search of medical databases, including PubMed, Web of Science, and Scopus, was performed to identify RCTs that assess the effects of probiotic and synbiotic supplementation on inflammatory markers in adult patients diagnosed with prediabetes and T2DM up to September 2025, with no restrictions imposed on time or language. The search was structured using the PICO (Participant, Intervention, Comparison/Control, Outcome) framework to ensure a focused selection of relevant studies. A mix of pertinent Medical Subject Headings (MeSH) and non-MeSH keywords was utilized, connected through Boolean operators (AND, OR). Searches were performed within the [Title/Abstract] fields to facilitate the precise retrieval of studies. The search strategy incorporated keywords such as ("probiotic∗" OR "prebiotic∗" OR "synbiotic∗") AND (“Inflammatory” OR “Inflammation” OR “Cytokines” OR “c-reactive protein” OR “CRP” OR “interleukin-6” OR “IL-6” OR “tumor necrosis factor” OR “tumor necrosis factor-alpha” OR “TNF” OR “TNF-α”) AND ("Diabetes" OR "Type 2 Diabetes Mellitus" OR "T2DM" OR "Diabetic"). The complete and exact search keywords and search lines are presented in the Supplemental Tables 1 and 2. Additionally, a manual review of reference lists from selected studies and an extended search on Google Scholar was conducted to identify further relevant studies.

### Eligibility criteria

The study's eligibility criteria were defined using the PICO framework to guarantee a systematic and focused selection of studies. Adults diagnosed with prediabetes or T2DM were deemed eligible participants. The intervention of interest involved probiotic or synbiotic supplementation, which was compared against placebo, control groups, or no treatment. Primary outcomes focused on changes in inflammatory markers, including CRP, IL-6, and TNF-α.

The exclusion criteria consisted of the following: *1*) participants who were not diabetic or prediabetic; *2*) studies focusing on interventions other than those specified were excluded; *3*) nonrandomized controlled trials, including animal studies, in vitro studies, case series, case-control studies, cohort studies, abstracts, letters, systematic reviews, and meta-analyses, were excluded; *4*) trials with follow-up durations of <1 wk postintervention were excluded; *5*) studies centered on irrelevant data; and *6*) studies with inaccessible full texts were also excluded. No restrictions were placed on the time frame, language, or participant demographics, including sex, age, ethnicity, or comorbidities. This approach facilitated a comprehensive yet targeted analysis. Such a rigorous methodology ensured that only studies of significant relevance and high quality were included, thoroughly addressing the research objectives.

### Data extraction

Two reviewers (AS and OA) performed data extraction independently, meticulously assessed the included studies, and collected all relevant information. Any disagreements or differences in the relevance or interpretation of data were resolved through discussions with a third reviewer (MK) to ensure accuracy and consensus. The extracted data have been methodically organized and systematically recorded in an Excel spreadsheet, providing clarity and consistency throughout the documentation process. The data gathered from each study included the first author’s name, year of publication, trial country, study design, sample size, mean age, BMI (in kg/m^2^), gender distribution, type of intervention, dosage, duration of intervention, and the overall health status of participants, and information on inflammatory markers like CRP, IL-6, and TNF-α was also included.

### Quality assessment

The Cochrane Collaboration tool [25] evaluated the quality of RCTs. All potential biases were assessed, including 5 domains: D1: randomization process; D2: deviations from the intended interventions; D3: missing outcome data; D4: measurement of the outcome; D5: selection of the reported result.

### Certainly evidence (Grading of Recommendations, Assessment, Development, and Evaluations assessment)

The certainty of evidence was assessed following the Grading of Recommendations, Assessment, Development, and Evaluations (GRADE) framework, which evaluates factors such as risk of bias, inconsistency, indirectness, imprecision, and potential publication bias. Each outcome was categorized into one of 4 certainty levels: high, moderate, low, or very low. This evaluation process involved analyzing study design, methodological quality, and the overall strength of the evidence to ensure a rigorous and transparent assessment of the findings [26].

### Statistical analysis

Statistical analyses were conducted using Stata version 11.0 (StataCorp), with a significance threshold of *P* < 0.05 for all 2-tailed tests. A random-effects model was employed to calculate pooled weighted mean differences (WMD), accounting for heterogeneity [27]. Mean differences in inflammatory markers were determined between baseline and postintervention for the intervention and control groups. The SD of mean differences was derived using the formula: SD = √[(SD at baseline)^2^ + (SD at the end)^2^ − (2 × *r* × SD at baseline × SD at the end)] [28], with a correlation coefficient (*r*) of 0.8. For studies reporting SE instead of SD, the Hozo et al. [29] method was used to convert SE, 95% confidence intervals (CIs), and IQRs into SDs. Heterogeneity was evaluated using Cochrane’s Q test and the I^2^ statistic, with significant heterogeneity defined as I^2^ >40%. Subgroup analyses were conducted based on diabetic status (prediabetic and T2DM), intervention type (probiotics vs. synbiotics), trial duration (<12 wk vs. ≥12 wk), and baseline BMI (normal, overweight, obese) [30,31]. Publication bias was assessed via visual inspection of funnel plots and Egger’s and Begg’s tests, with sensitivity analyses performed using the leave-one-out method [32,33]. Metaregression and nonlinear dose–response regression were employed to assess the effects of intervention dose and duration on inflammatory markers and to synthesize correlated dose–response data across studies [34,35]. The trim-and-fill method was applied to address publication bias [36].

## Results

### Study selection

**FIGURE 1:**
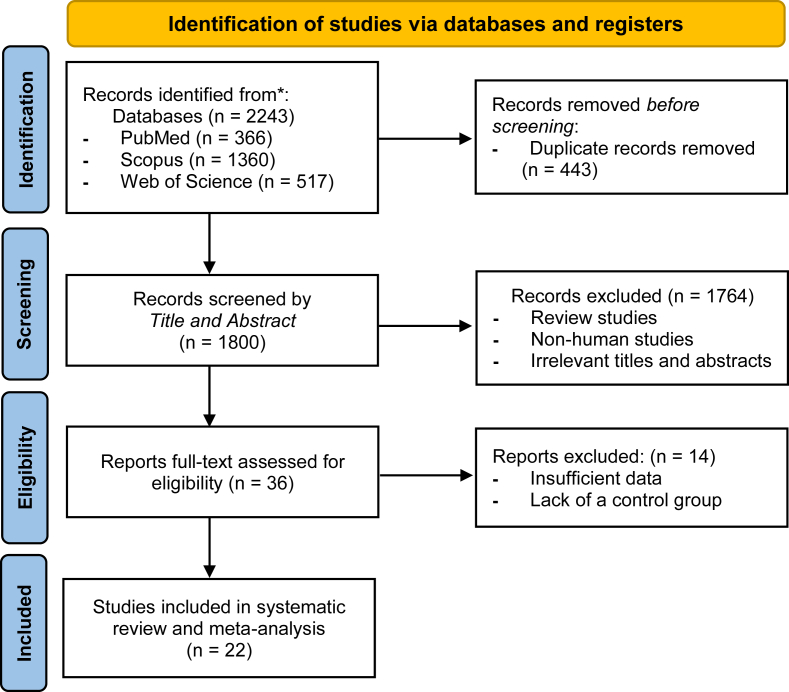
PRISMA flow chart of study selection process in the systematic review.

A comprehensive search was conducted across 3 databases, PubMed (*n* = 366), Web of Science (*n* = 517), and Scopus (*n* = 1360), resulting in a total of 2243 records. After the removal of 443 duplicate entries, the remaining 1800 studies underwent a thorough screening of their titles and abstracts. During this process, 1764 articles were excluded because they were deemed irrelevant to the research topic. The full texts of the remaining 36 studies were then meticulously evaluated for eligibility, leading to the exclusion of 14 studies that lacked the necessary data for analysis. As a result, a total of 22 eligible RCTs were included in this meta-analysis [[37], [38], [39], [40], [41], [42], [43], [44], [45], [46], [47], [48], [49], [50], [51], [52], [53], [54], [55], [56], [57], [58]], providing valuable insights for the study. The selection process is visually represented in Figure 1.

### Study characteristics

A total of 22 RCTs [[37], [38], [39], [40], [41], [42], [43], [44], [45], [46], [47], [48], [49], [50], [51], [52], [53], [54], [55], [56], [57], [58]] with 24 effect sizes assessed inflammatory markers. Of these, 17 effect sizes were reported for CRP [[38], [39], [40], [41], [42], [43],[45], [46], [47], [48],[50], [51], [52], [53],^55^,^56^], 14 for IL-6 [37,39,[42], [43], [44], [45], [46],[48], [49], [50], [51], [52],^55^,^58^], and 14 for TNF-α [37,42,44,45,[48], [49], [50], [51], [52],^54^,^55^,^57^,^58^]. The study design characteristics presented in Table 1 [[37], [38], [39], [40], [41], [42], [43], [44], [45], [46], [47], [48], [49], [50], [51], [52], [53], [54], [55], [56], [57], [58]] indicate that the studies included in this analysis were conducted between 2013 and 2025. In our analysis, 1321 participants diagnosed with prediabetes and T2DM were included, comprising 693 individuals in the intervention group and 628 individuals in the control group.

**TABLE 1:** Characteristics of the included studies in the systematic review and meta-analysis.

Studies	Country	Study design	Participant	Gender	Sample size		Trial duration (wk)	Means age		Means BMI		Intervention		
					IG	CG		IG	CG	IG	CG	Type	Dose	Control group
Mazloom et al. [46], 2013	Iran	Parallel, R, PC, DB	T2DM	M/F (8, 26)	16	18	6	55.4 ± 8	51.8 ± 10.2	27.97±3.81	27.24 ± 2.73	Probiotic (multistrain)	1500 mg/d	Magnesium stearate
Kooshki et al. [45], 2015	Iran	Parallel, R, PC, DB	T2DM	M/F (16, 28)	22	22	8	53.45 ± 10.8	54.5 ± 11.10	22.79±2.7	22.47 ± 2.38	Synbiotic	1 tablet/d	Placebo
Firouzi et al. [40], 2017	Malaysia	Parallel, R, PC, DB	T2DM	M/F (65, 71)	68	68	12	52.9 ± 9.2	54.2 ± 8.3	29.2 ± 5.6	29.3 ± 5.3	Probiotic (multistrain)	6×10^10^ CFU/d	Placebo
Mobini et al. [47], 2017 (B)	Sweden	Parallel, R, PC, DB	T2DM	M/F (17, 5)	14	8	12	64 ± 6	65 ± 5	32.3 ± 3.4	30.7 ± 4	Probiotic *(L. Reuteri* DSM 17938) (high dose)	10^10^ CFU/d	Placebo
Mobini et al. [47], 2017 (A)	Sweden	Parallel, R, PC, DB	T2DM	M/F (17, 5)	15	7	12	66 ± 6	65 ± 5	30.6 ± 4.5	30.7 ± 4	*Probiotic (L. Reuteri* DSM 17938 (low dose)	10^8^ CFU/d	Placebo
Ahmadian et al. [37], 2017	Iran	Parallel, R, PC, DB	T2DM	M/F (29, 30)	30	29	6	58.5 ± 3.25	61 ± 2	27.4 ± 4	27.16 ± 4	Synbiotic	10^9^ CFU/g and 100 mg FOS	Magnesium stearate
Hsieh et al. [42], 2018	Taiwan	Parallel, R, PC, DB	T2DM	M/F (25, 19)	22	22	24	52.32 ± 10.20	55.77 ± 8.55	28.04±4.29	27.53 ± 3.15	Probiotic (*L. Ruteri*. Live)	2 × 10^9^ CFU/capsule	Placebo
Kobyliak et al. [44], 2018	Ukraine	Parallel, R, PC, DB	T2DM	M/F	31	22	8	52.23 ± 1.74	57.18 ± 2.06	34.7 ± 1.29	35.65 ± 1.57	Probiotic (multistrain) (Symbiter)	10 g/d	Placebo
Sabico et al. [48], 2019	Saudi Arabia	Parallel, R, PC, DB	T2DM	M/F (40, 38)	31	30	24	48 ± 8.3	46.6 ± 5.9	29.4 ± 5.2	30.1 ± 5	Probiotic (multistrain)	2.5 × 10^9^ CFU/g	Placebo
Tay et al. [50], 2020	New Zealand	Parallel, R, PC, DB	Prediabetic	M/F (8, 18)	15	11	12	52.9 ± 8.7	54.1 ± 6.4	34.7 ± 4.9	33.6 ± 3.7	Probiotic *(L. Rhamnosus* Hnoo1)	6 × 10^9^ CFU	Microcrystalline cellulose and dextrose anhydrate
Velayati et al. [53], 2021	Iran	Parallel, R, PC, DB	T2DM	M/F (17, 26)	20	23	12	59.10 ± 9.71	60.39 ± 6.74	27.32±4.34	28.27 ± 2.54	Synbiotic (FOS)	1 sachet/d	Starch + 0.7% natural orange flavor
Toejing et al. [52], 2021	Thailand	Parallel, R, PC, DB	T2DM	M/F (18, 18)	18	18	12	63.5 ± 5.94	61.78 ± 7.73	23.22±2.72	23.05 ± 2.6	Probiotic (*L. Paracasei* Hii01)	50 × 10^9^ CFU/d	Placebo
Kanazawa et al. [43], 2021	Japan	Parallel, R, C,	T2DM	M/F (65, 21)	44	42	24	61.1 ± 11.0	55.9 ± 10.7	29.5 ± 4.4	29.1 ± 3.7	Synbiotic	Mixture 10.5 g/d	Placebo
Wei et al. [54], 2022	China	Parallel, R, PC, DB	Depression and diabetes	M/F (33, 27)	30	30	1	43.59 ± 4.32	43.31 ± 4.57	23.37±1.31	23.33 ± 1.24	Compound lactic acid bacteria capsules combined with escitalopram	3 times/d at a dose of 0.66 g/time	Escitalopram
Eliuz et al. [39], 2023	Turkey	Parallel, R, PC	T2DM	F (34)	17	17	8	45.0 ± 6	48 ± 6.75	33.5 ± 5.77	31.1 ± 4.82	Probiotic	10×10^9^ CFU/d *Lactobacillus rhamnosus* GG ATCC 53,103 (LGG)	Corn oil
Savytska et al. [49], 2023	Ukraine	Parallel, R, PC, DB	T2DM	M/F	34	34	8	53.82 ± 9.58	56.93 ± 9.88	31.92±4.96	32.57 ± 6.16	Probiotic (multistrain)	1 pack (10 g)	Placebo
Yarahmadi et al. [55], 2024	Iran	Parallel, R, PC, DB	T2DM with periodontal disease	M/F (33, 14)	23	24	8	48.6 ± 5.8	50.1 ± 3.6	24 ± 3.6	25.5 ± 2.7	Multispecies probiotic + 100 mg FOS	Multispecies *Lactobacillus acidophilus* + 100 mg FOS	Placebo
Zhang et al. [58], 2024	China	Parallel, R, DB	T2DM	M/F (12, 8)	10	10	4	62.6 ± 10.96	60.6 ± 11.16	23.11±3.42	25.783 ± 1.774	*Bifidobacterium bifidum*	1.5 g/times, 3 times/d	Standard Western medical treatments
Tian et al. [51], 2025	China	Parallel, R, PC, DB	T2DM	M/F (35, 25)	30	30	12	49.77	49.81	25.98±0.76	25.91 ± 0.72	Dulaglutide 1.5 mg/wk + probiotics containing *Bifidobacterium longum*, 2 × 10^9^ CFU/dose	Dulaglutide 1.5 mg/wk + probiotics containing *Bifidobacterium longum*, 2 × 10^9^ CFU/dose	Dulaglutide 1.5 mg/wk
Chaithanya et al. [38], 2025	India	Parallel, R, PC, DB	T2DM	M/F (51, 79)	65	65	24	55.0 ± 9.16	57.6 ± 8.56	27.3 ± 4.13	28.6 ± 5.04	Lactobacillus, Bifidobacterium, *Streptococcus thermophilus*, and *Saccharomyces boulardii*	3 × 10^10^ CFUs	Placebo
Yazdani et al. [56], 2025	Iran	Parallel, R, PC, DB	T2DM and fatty liver	M/F (10, 30)	20	20	12	56.75	55.5	29.62	29.99	Multistrain synbiotic	21 mg FOS	Maltodextrin
Geng et al. [41], 2025	China	Parallel, R, PC, DB	T2DM	M/F (42, 34)	38	38	12	59.55	54.68	25.61	25.35	*Lactobacillus rhamnosus* LRa05	LRa05 (0.1 g 2 × 10^10^ CFU)	Placebo
Zhang et al. [57], 2025 (A)	China	Parallel, R, PC, DB	T2DM	M/F (20, 40)	40	20	12	58	61	25	25	*Bifidobacterium animalis* subsp. lactis MN-Gup	0.1 g of MN-Gup powder, delivering 5 × 10^10^ CFU along with 3.4 g of maltodextrin;	3.5 g of maltodextrin
Zhang et al. [57], 2025 (B)	China	Parallel, R, PC, DB	T2DM	M/F (20, 40)	40	20	12	58	61	24.7	25	Synbiotic mixture of MN-Gup and GOS	0.1 g of MN-Gup powder with 5 × 10^10^ CFU of viable bacteria, 0.9 g of GOS, and 2.5 g of maltodextrin	3.5 g of maltodextrin

Abbreviations: CFU, colony-forming unit; CI, confidence interval; FOS, fructooligosaccharide; GOS, galactooligosaccharide; RCT, randomized controlled trials; T2DM, type 2 diabetes mellitus; WMD, weighted mean differences; PC, Placebo-controlled, DB, double-blinded, IG, intervention group, CG, control group.

The research was conducted in various countries, with a significant proportion in Iran [37,45,46,53,55,56] and China [41,51,54,57,58]. Other nations involved in this research include India [38], Japan [43], New Zealand [50], Taiwan [42], Sweden [47], Malaysia [40], Ukraine [44,49], Thailand [52], Japan [43], China [54], Turkey [39], and Saudi Arabia [48]. All included RCTs were conducted using a parallel, double-blind design. The mean age and baseline BMI of the participants in the studies considered ranged from 43 to 66 y and 22.79 to 34.7 in the intervention group, respectively. The duration of supplementation reported in the studies varied from 1 wk to 24 wk. Except for 1 study that exclusively involved female subjects [39]. All studies included both male and female subjects [37,38,[40], [41], [42], [43], [44], [45], [46], [47], [48], [49], [50], [51], [52], [53], [54], [55], [56], [57], [58]].

All studies reported on patients with diabetes [[37], [38], [39], [40], [41], [42], [43], [44], [45], [46], [47], [48], [49],[51], [52], [53], [54], [55], [56], [57], [58]], except 1 study that included prediabetic subjects [50]. In the studies conducted by Mobini et al. [47] and Zhang et al. [57], we considered 2 arms for this study, based on the application of high-dose and low-dose interventions, as well as probiotic and synbiotic interventions.

### Meta-analysis on CRP

**FIGURE 2:**
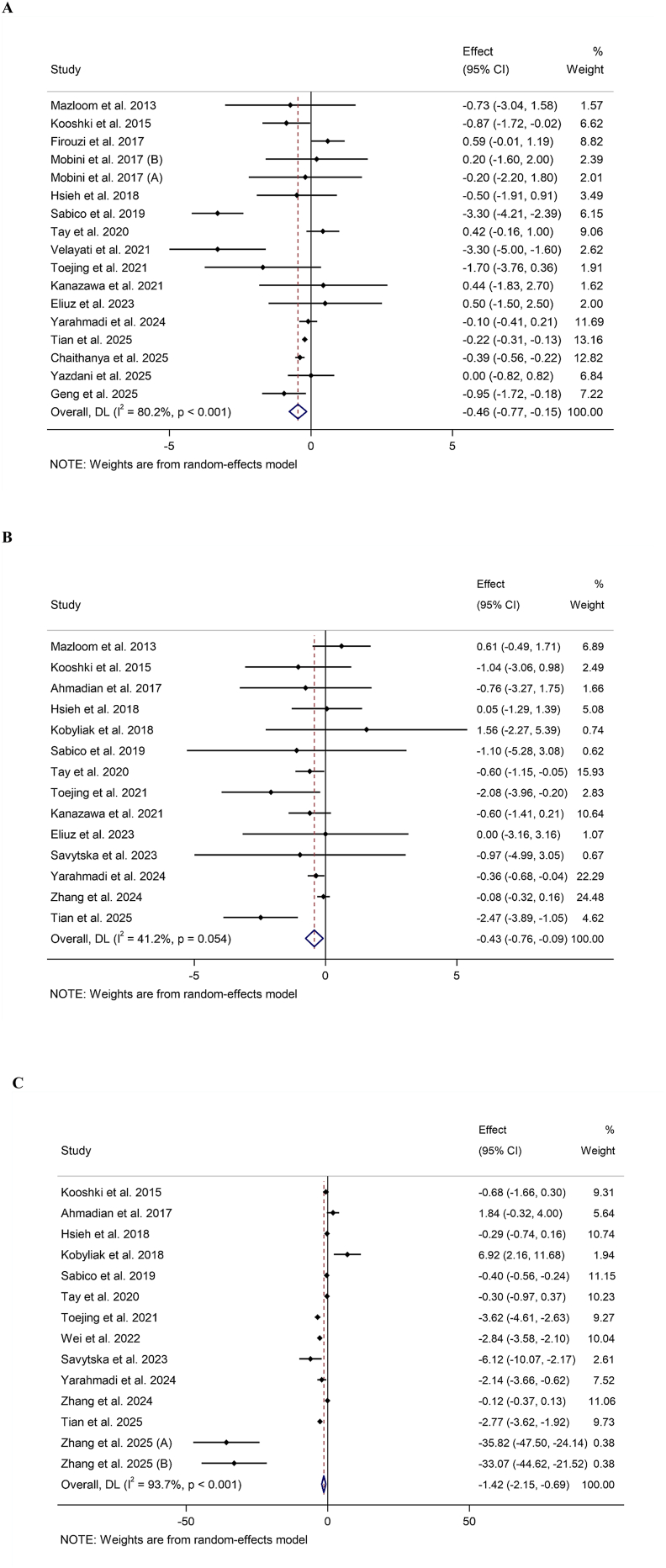
Forest plot demonstrating mean difference (MD) and 95% confidence intervals (CIs) for the effect of probiotic and synbiotic supplementation on (A) C-reactive protein (CRP) (mg/L), (B) IL-6 (pg/mL), and (C) TNF-α (pg/mL).

An analysis that pooled data from 17 RCTs [[38], [39], [40], [41], [42], [43],[45], [46], [47], [48],[50], [51], [52], [53],^55^,^56^], which included a total of 941 participants (478 receiving probiotics and synbiotics and 463 in the control group), found that supplementation significantly decreased levels of CRP. The observed WMD was −0.46 mg/L, with a 95% CI of −0.77 to −0.15. Furthermore, considerable heterogeneity was noted (I^2^ = 80.2%, *P* < 0.001) (Figure 2A, Table 2).

**TABLE 2:** Meta-analysis findings of probiotics and synbiotics supplementation on inflammatory cytokines in adults with prediabetes and T2DM.

	No. of ES	WMD (95% CI)	*P* value	Heterogeneity	
				P-heterogeneity	I^2^ (%)
C-reactive protein (CRP) (mg/L)					
Overall effect	17	−0.46 (−0.77, −0.15)	0.003^1^	<0.001	80.2
CRP baseline					
≥3 (mg/L)	11	−0.81 (−1.34, −0.28)	0.002^1^	<0.001	85.7
<3 (mg/L)	5	0.00 (−0.53, 0.54)	0.973	0.035	61.3
Trial duration (wk)					
<12	4	−0.25 (−0.66, −0.16)	0.239	0.327	13.2
≥12	13	−0.51 (−0.89, −0.14)	0.006^1^	<0.001	84.5
Diabetic status					
T2DM	16	−0.54 (−0.86, −0.22)	0.001^1^	<0.001	80.1
Prediabetic	1	0.42 (−0.15, 0.99)	0.154	—	—
Intervention type					
Probiotic	12	−0.44 (−0.81, −0.07)	0.020^1^	<0.001	83.1
Synbiotic	5	−0.63 (−1.44, 0.18)	0.128	0.003	74.9
Baseline BMI (kg/m^2^)					
Normal (<25)	3	−0.54 (−1.31, 0.22)	0.162	0.090	58.4
Overweight (25–29.9)	10	−0.65 (−1.07, −0.24)	0.002^1^	<0.001	87.1
Obese (>30)	4	0.36 (−0.14, 0.88)	0.161	0.942	0.0
IL-6 (pg/mL)					
Overall effect	14	−0.43 (−0.76, −0.09)	0.012^1^	0.054	41.2
Trial duration (wk)					
<12	8	−0.16 (−0.35, 0.02)	0.084	0.577	0.0
≥12	6	−0.92 (−1.59, −0.25)	0.007^1^	0.094	46.8
Diabetic status					
T2DM	13	−0.40 (−0.78, −0.02)	0.035^1^	0.057	41.8
Prediabetic	1	−0.60 (−1.14, −0.05)	0.032^1^	—	—
Intervention type					
Probiotic	10	−0.47 (−1.04, 0.10)	0.106	0.018	55.1
Synbiotic	4	−0.41 (−0.70, −0.11)	0.006^1^	0.860	0.0
Baseline BMI (kg/m^2^)					
Normal (<25)	4	−0.31 (−0.71, 0.07)	0.115	0.092	53.4
Overweight (25–29.9)	6	−0.58 (−1.52, 0.34)	0.217	0.033	58.8
Obese (>30)	4	−0.54 (−1.07, −0.01)	0.043^1^	0.716	0.0
TNF-α (pg/mL)					
Overall effect	14	−1.42 (−2.15, −0.69)	<0.001^1^	<0.001	93.7
Trial duration (wk)					
<12	7	−0.82 (−2.26, 0.62)	0.266	<0.001	91.8
≥12	7	−2.17 (−3.44, −0.89)	0.001^1^	<0.001	95.5
Diabetic status					
T2DM	13	−1.55 (−2.35, −0.76)	<0.001^1^	<0.001	94.2
Prediabetic	1	−0.30 (−0.97, 0.37)	0.383	—	—
Intervention type					
Probiotic	10	−1.49 (−2.27, −0.71)	<0.001^1^	<0.001	94.6
Synbiotic	4	−2.80 (−6.39, 0.77)	0.125	<0.001	92.2
Baseline BMI (kg/m^2^)					
Normal (<25)	6	−2.53 (−4.31, −0.74)	0.006^1^	<0.001	95.8
Overweight (25–29.9)	5	−1.03 (−2.32, 0.25)	0.115	<0.001	94.2
Obese (>30)	3	−0.02 (−5.33, 5.28)	0.993	<0.001	88.4

Abbreviations: CI, confidence interval; ES, effect size; T2DM, type 2 diabetes mellitus; WMD, weighted mean differences.

1 Statistically significant [p < 0.05].

Subgroup analyses based on baseline CRP levels, trial duration, and type of intervention demonstrated a significant decrease in CRP levels for those with baseline CRP levels ≥3, consistent across studies with trial duration (<12 wk; ≥12 wk). The analysis showed the beneficial effects associated with consuming probiotics. In patients with elevated CRP (≥3 mg/L), additionally, participants diagnosed with T2DM demonstrated a substantial decrease in CRP levels compared with prediabetic subjects. A further subgroup analysis based on baseline BMI indicated a noteworthy decline in CRP levels among participants classified as overweight (BMI: 25–29.9) (Table 2).

### Meta-analysis on IL-6

The RCTs included in this meta-analysis were 14 [37,39,[42], [43], [44], [45], [46],[48], [49], [50], [51], [52],^55^,^58^]. As shown in Figure 2B, the overall WMD is −0.43 pg/mL, with a 95% CI ranging from −0.76 to −0.09. This outcome indicates low heterogeneity, as evidenced by I^2^ = 41.2% (*P* = 0.054). Furthermore, subgroup analyses indicated a statistically significant reduction in IL-6 levels among studies involving participants with obesity who underwent treatment for durations longer than 12 wk and when synbiotics were used as an intervention (Table 2).

### Meta-analysis on TNF-α

Fourteen RCTs investigated the change in the mean TNF-α in patients after the intervention [37,42,44,45,[48], [49], [50], [51], [52],^54^,^55^,^57^,^58^]. The combined analysis of these studies indicated a WMD of −1.42 pg/mL; 95% CI: −2.15, −0.69 (Figure 2C). The subgroup analysis included trial duration, health status, and baseline BMI, which has shown a significant decrease in studies with a trial duration of 12 wk or more, specifically among subjects with T2DM and a baseline BMI classified as normal (<25), and when probiotic was used as an intervention (Table 2).

### Nonlinear dose–response analysis

**FIGURE 3:**
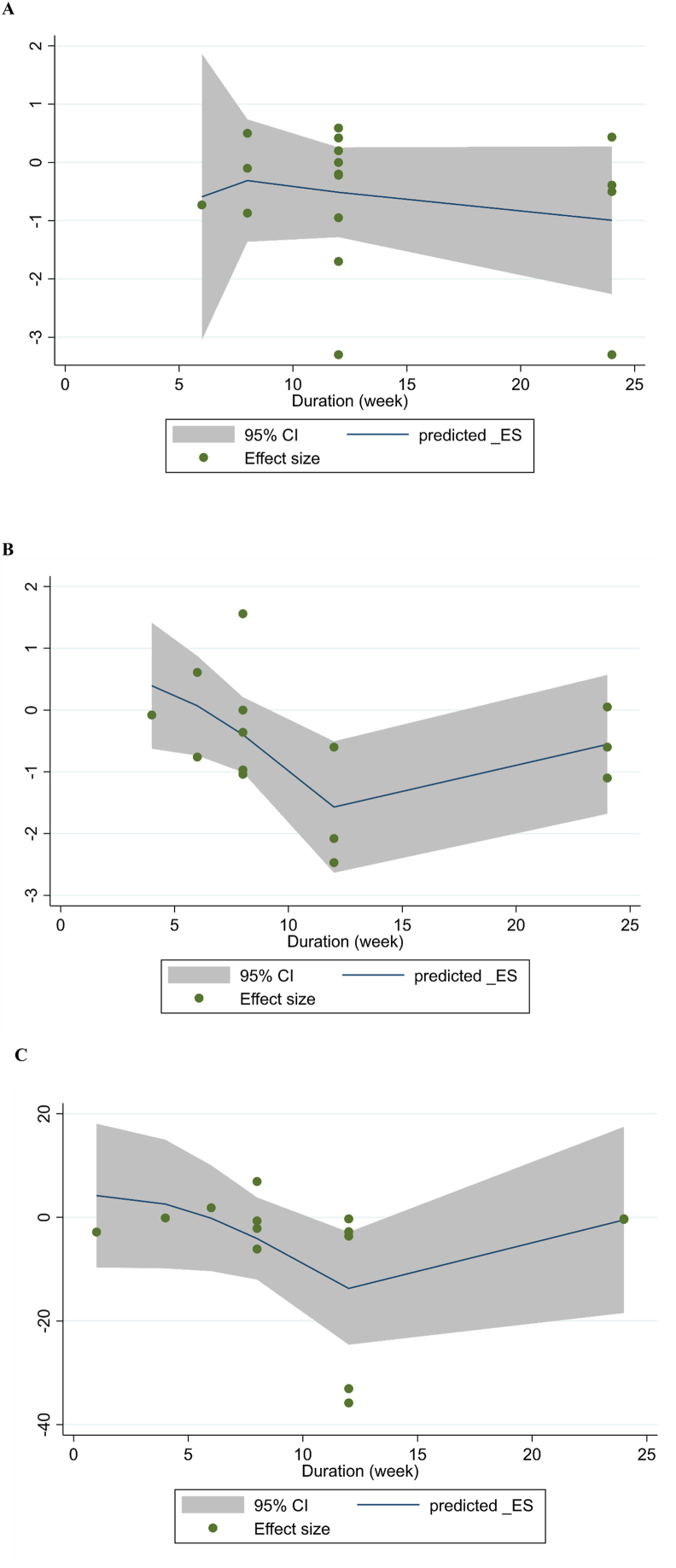
Nonlinear dose–response relations between duration of intervention (wk) and absolute mean differences in (A) C-reactive protein (CRP), (B) IL-6, and (C) TNF-α.

A thorough analysis of the nonlinear dose–response relationship based on the duration of the intervention revealed a significant relationship between the duration of the intervention and changes in IL-6 levels (*P* = 0.029). However, there was no significant relationship with changes in CRP levels (*P* = 0.476) or TNF-α levels (*P* = 0.065) (Figure 3) (Supplemental Table 3).

### Metaregression analysis

**FIGURE 4:**
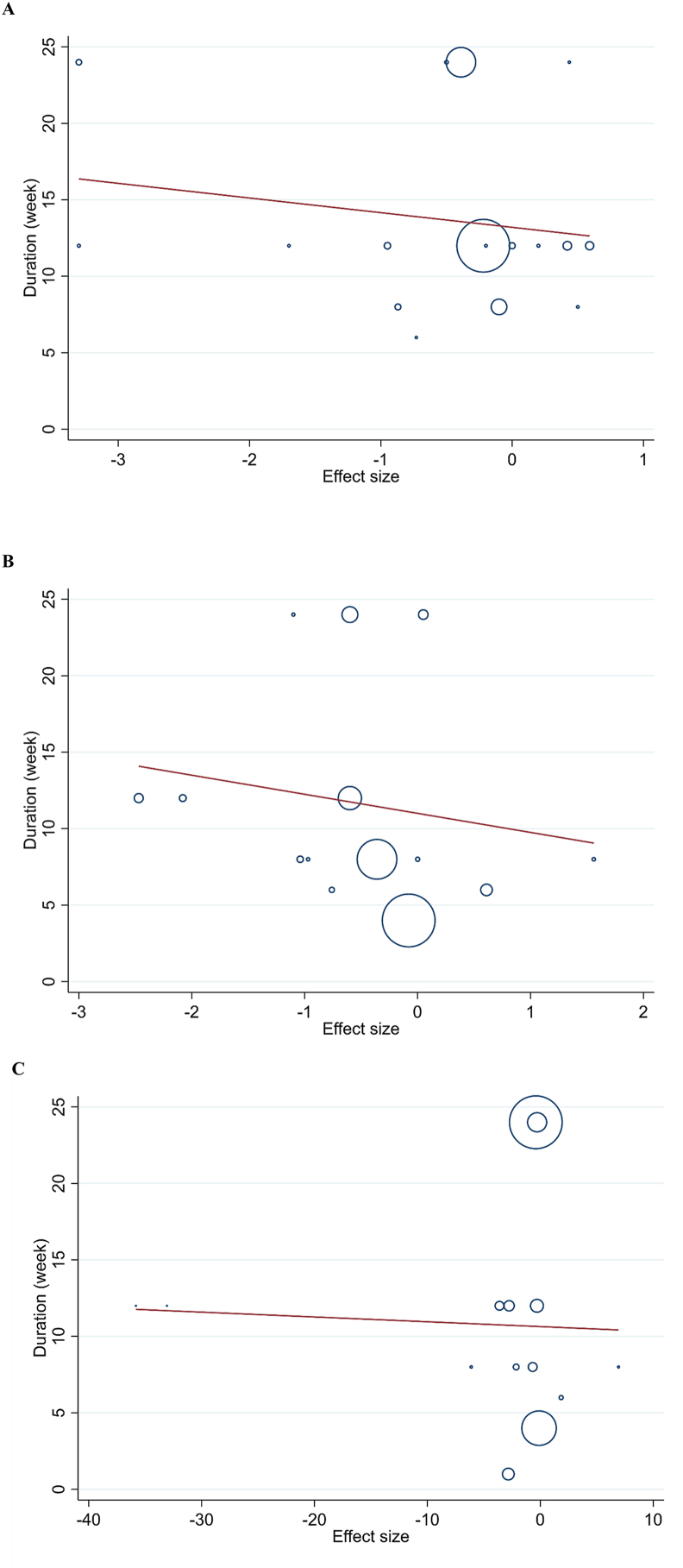
Linear dose–response relations between duration of intervention (wk) and absolute mean differences in (A) C-reactive protein (CRP), (B) IL-6, and (C) TNF-α.

A linear metaregression analysis was conducted to investigate the association between CRP, IL-6, and TNF-α levels and the duration of probiotic and synbiotic supplementation. The findings of this metaregression revealed no correlation between the duration of the trials and changes in CRP (*P* = 0.480), IL-6 (*P* = 0.539), and TNF-α (*P* = 0.873) (Figure 4) (Supplemental Table 3).

### Publication bias and sensitivity analysis

**FIGURE 5:**
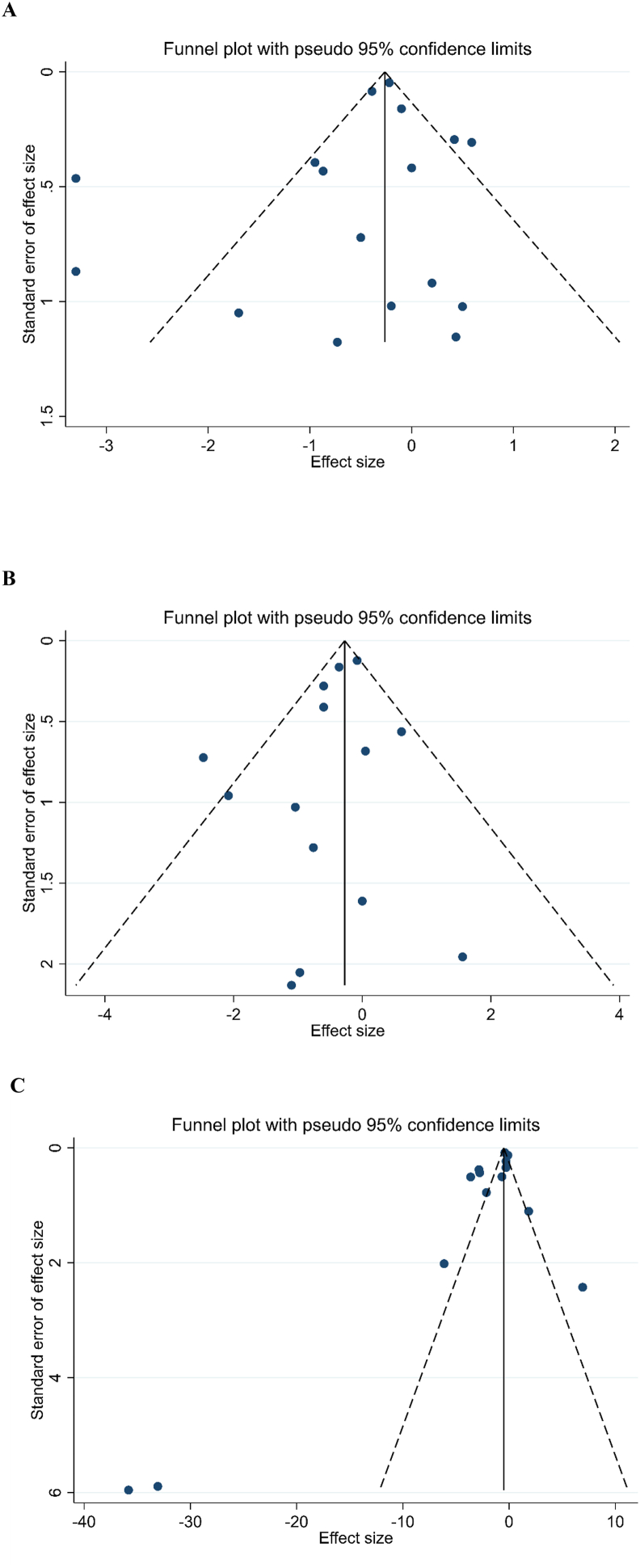
Funnel plots of publication bias for the effect of the probiotic and synbiotic supplementation on (A) C-reactive protein (CRP), (B) IL-6, and (C) TNF-α.

The publication bias assessment showed no significant bias for most variables, as indicated by nonsignificant Egger’s and Begg’s tests, except for TNF-α, which had significant Egger’s test results (*P* = 0.031) (Figure 5, Supplemental Table 4).

### Sensitivity analysis

The sensitivity analysis revealed that no individual study had a significant influence on the overall results for CRP, IL-6, and TNF-α. (Supplemental Table 4).

### Risk of bias assessment 2

The risk of bias assessment 2 revealed that 9 of 22 studies were classified as "Fair." Additionally, 9 studies were classified as "Bad," and 4 studies were classified as "Good" (Table 3) [[37], [38], [39], [40], [41], [42], [43], [44], [45], [46], [47], [48], [49], [50], [51], [52], [53], [54], [55], [56], [57], [58]].

**TABLE 3:** Risk of bias assessment (R0B-V2).

Studies	D1	D2	D3	D4	D5	Overall
Mazloom et al. [46], 2013	L	L	H	L	H	Fair
Kooshki et al. [45], 2015	L	L	L	L	L	Good
Firouzi et al. [40], 2017	L	L	L	L	H	Fair
Mobini et al. [47], 2017	L	L	H	L	H	Bad
Ahmadian et al. [37], 2017	L	L	H	L	H	Bad
Hsieh et al. [42], 2018	L	L	L	L	L	Good
Kobyliak et al. [44], 2018	L	L	H	L	H	Bad
Sabico et al. [48], 2019	L	L	H	L	L	Fair
Tay et al. [50], 2020	L	L	H	L	L	Fair
Velayati et al. [53], 2021	L	L	H	L	H	Bad
Toejing et al. [52], 2021	L	L	L	L	L	Good
Kanazawa et al. [43], 2021	L	H	H	L	H	Bad
Wei et al. [54], 2022	H	L	L	L	H	Bad
Eliuz et al. [39], 2023	L	H	L	L	H	Bad
Savytska et al. [49], 2023	L	L	L	L	H	Fair
Yarahmadi et al. [55], 2024	L	L	H	L	L	Fair
Zhang et al. [58], 2024	L	H	L	L	H	Bad
Tian et al. [51], 2025	L	L	L	L	L	Good
Chaithanya et al. [38], 2025	L	L	L	L	H	Fair
Yazdani et al. [56], 2025	L	L	L	L	H	Fair
Geng et al. [41], 2025	L	L	L	L	H	Fair
Zhang et al. [57], 2025	L	L	H	L	H	Bad

Domains: D1: randomization process; D2: deviations from the intended interventions; D3: missing outcome data; D4: measurement of the outcome; D5: selection of the reported result.

Interpretation of overall risk of bias: bad > 1 high risk; fair = 1 high risk; good < 1 high risk.

### GRADE certainly assessment

The GRADE evaluation of the study indicated that the quality of evidence for CRP and TNF-α was moderate, and for IL-6, it was high. With significant limitations due to a high level of heterogeneity. Additionally, no serious limitations regarding the risk of bias, indirectness, or imprecision were noted for any outcome (Table 4).

**TABLE 4:** GRADE profile of evidence assessment of probiotics and synbiotics supplementation on inflammatory cytokines in individuals with prediabetes and type 2 diabetes mellitus.

Outcomes	Risk of bias	Inconsistency	Indirectness	Imprecision	Publication bias	Quality of evidence
CRP	No serious limitation	Very serious limitation^1^	No serious limitation	No serious limitation	No serious limitation	⊕⊕◯◯ Moderate
IL-6	No serious limitation	Serious limitation^2^	No serious limitation	No serious limitation	No serious limitation	⊕⊕⊕◯ High
TNF-α	No serious limitation	Very serious limitation^1^	No serious limitation	No serious limitation	No serious limitation	⊕⊕◯◯ Moderate

Abbreviations: CRP, C-reactive protein; GRADE, Grading of Recommendations, Assessment, Development, and Evaluations.

1 There is a high level of heterogeneity (I^2^>75).

2 There is a moderate level of heterogeneity (I^2^>40).

## Discussion

### Aim and main findings

T2DM is a metabolic disease characterized by a chronic inflammatory phenomenon. Several studies revealed that systemic inflammatory markers accelerate the progression of diabetes and trigger subsequent complications [59,60]. Alteration in gut microbiota affects inflammatory response, oxidative stress, and glucose and lipid metabolism, thus playing a critical role in the pathogenesis of T2DM [61]. Probiotics and synbiotics reverse the pathogenic dysbiosis of the gut microbiota and play a favorable role in mitigating inflammatory activities [62]. Therefore, this meta-analysis aimed to elucidate the efficacy of probiotic and synbiotic supplements in reducing inflammatory markers and delaying or preventing disease progression in patients with prediabetes and T2DM.

The findings of this meta-analysis suggest that probiotic and synbiotic supplementation significantly reduces CRP, IL-6, and TNF-α as the inflammatory markers. Subgroup analyses revealed that CRP reduction was most pronounced in individuals with a baseline CRP level of ≥3 mg/L, those with longer intervention durations (≥12 wk), those with T2DM and overweight patients, and when probiotics were used as an intervention. IL-6 levels were significantly reduced in individuals with obesity with longer treatment durations and when synbiotics were used as an intervention. TNF-α reduction was most evident in long-term interventions (≥12 wk) among patients with T2DM with normal BMI and when probiotics were used as an intervention.

### Underlying mechanism

The key benefit of probiotics and synbiotics lies in their ability to establish a suitable balance between pathogenic microorganisms and essential bacteria, thereby impacting the development of the host microbiome [63]. The mechanism underlying this modulating effect is not yet fully understood; however, earlier studies in molecular and genetic research explain the 4 main mechanisms responsible for the beneficial effects: *1*) production of SCFA molecules by the intestinal microbiome reduces the production of CRP marker in the liver [64]; *2*) the effect of probiotic and synbiotic supplements on improving lipid profile restricts inflammatory activities [65]; *3*) suppression of the nuclear factor kappa B signaling pathway by controlling blood glucose level and preventing a hyperglycemic state that subsequently decreases the serum level of IL-6, which reduces the expression of the CRP genes [66,67]; and *4*) the antioxidative effect of these supplements that downregulates both inflammation and oxidative stress [68]. However, further investigation is necessary to elucidate the potential mechanisms underlying the effect of probiotic and synbiotic supplements on inflammation in patients with T2DM. Additionally, special attention is needed for the prediabetic population to prevent the development of T2DM in its early stages.

### Comparison with studies

The current meta-analysis revealed a significant effect of probiotics and synbiotics on reducing the level of the CRP marker in patients with prediabetes and T2DM (WMD: −0.46; 95% CI: −0.76, −0.15). Similar findings from 2 other meta-analyses showed a notable decrease in hs-CRP and CRP levels after administering probiotics and synbiotics compared with the placebo treatment [21,22]. However, those studies only focused on patients with diabetes, whereas our research targeted prediabetes and T2DM. Results from our analyses regarding serum TNF-α levels were quite similar to those of CRP. A favorable effect of probiotic and synbiotic supplements on TNF-α levels has been reported. In line with our research, a meta-analysis detected notable reductions in TNF-α after consuming daily doses of multistrain probiotics [69]. Another study reported that supplements containing *Lactobacillus plantarum* could increase insulin sensitivity by restricting the production of inflammatory markers, especially TNF-α [70]. Concerning IL-6 levels, a decrease in serum levels was observed after prebiotic and synbiotic supplementation (WMD: −0.42; 95% CI: −0.76, −0.09). Previous studies were not entirely consistent with our results. Two other meta-analyses have reported no significant changes in serum IL-6 concentration after the intake of supplements [69,71]. The inconsistency in the findings could be attributed to the limited number of studies included in previous research regarding IL-6 levels, compared with our current meta-analysis.

### Subgroup analysis’ findings

Subgroup analysis for CRP and TNF-α levels revealed a significant effect was pronounced in those with a definite diagnosis of T2DM and not in the patients with prediabetes. However, a beneficial effect on IL-6 was demonstrated in both T2DM and prediabetic groups. Also, this study has revealed a strong relationship between supplementation and lowering the CRP level in patients with high-grade baseline inflammation (CRP level ≥3) compared with those with limited inflammatory status. These results support the notion that the benefits of probiotics and synbiotics are more substantial in individuals with heightened inflammation [67]. Regarding the duration of treatment, the mounting positive effects of these supplements were noted during longer trials (<12 wk and more) for all markers.

The subgroup analysis on BMI revealed that overweight and obesity are associated with a reduced response to the most favorable effects of the supplementation, particularly in terms of suppressing CRP and IL-6 markers. Previous investigations have mentioned that the products of excessive adipose tissue in patients with obesity might activate an "obesity-related inflammatory" state and trigger cardiometabolic comorbidities, thus mitigating the efficacy of anti-inflammatory treatment strategies [72,73]. This finding has the potential to be incorporated into future clinical guidelines for the prevention and management of diabetes.

### Clinical implications

Probiotics and synbiotics are natural residents of a healthy intestine. Certain conditions, such as illness, stress, and the use of antibiotics, can disrupt the normal flora. In these cases, probiotic and synbiotic supplements could help rebalance the microbiome [74,75]. Several studies reported the beneficial effects of probiotics from food or supplements on improving the inflammatory state of T2DM. Daily intake of probiotic-rich food is considered “good” and “safe.” However, to date, no established guideline exists for prescribing probiotics and synbiotics for diabetes management [76]. Also, the *Food and Drug Administration* (FDA) does not oversee the supplements, so the efficacy and dosage are unadjusted [77]. Therefore, on the basis of our findings, we propose that the FDA revise the current dietary guidelines for T2DM to include detailed recommendations for physicians and nutrition specialists on incorporating probiotic and synbiotic supplements, including precise dosage specifications and administration protocols.

### Strengths and limitations

Our study has several notable strengths. We exclusively extracted data from RCTs to gather high-quality evidence from a large target population. In contrast to the majority of earlier meta-analyses, which primarily focused on patients with diabetes, our study was conducted on both diabetic and prediabetic groups. The data were extracted, analyzed collectively, and further stratified separately for each subgroup. These insights pave the way not only for enhancing treatment strategies but also for preventing the onset of diabetes. Moreover, we performed a detailed examination of each inflammatory marker within various subgroups, including trial duration, participants' BMI categories, dose–response relationships, and the type of supplementation, whether it involved probiotics or synbiotics. In addition to enhancing the quality of the study and the level of evidence, we carried out a grading and assessment process. However, a limitation was the observed heterogeneity regarding the CRP and TNF-α markers. Therefore, it is essential to interpret these findings with caution. Altogether, this study offers valuable references for subsequent related studies and future clinical trials. Further studies are vital to thoroughly explore the optimal combination of probiotic strains and supplementation dosage for diabetes prevention and treatment.

## Conclusion

This systematic review and meta-analysis demonstrate that probiotic and synbiotic supplementation effectively reduces inflammatory markers, including CRP, IL-6, and TNF-α, in adults with prediabetes and T2DM. The most significant reductions in CRP were observed in individuals with higher baseline CRP levels, longer intervention durations, and those with T2DM, suggesting stronger anti-inflammatory effects in those with chronic inflammation. Similarly, IL-6 levels decreased significantly in individuals with T2DM and prediabetes with interventions lasting over 12 wk. TNF-α reduction was most notable in long-term interventions (≥12 mo) in individuals with T2DM and normal BMI. Despite some variability in results, the consistency of findings across subgroup analyses supports the potential therapeutic benefits of probiotics and synbiotics in mitigating inflammation associated with metabolic disorders.

## Author contributions

The authors’ responsibilities were as follows – OA, MK: conceived the study and designed the research methodology; AS, OA, MK: conducted the systematic review and data extraction; OA, MK: performed the statistical analysis and interpreted the results; MK, OA, FV, SP: contributed to the drafting of the manuscript; MK, OA, SHD, BL: reviewed, conceptualized, and supervised the project; and all authors: critically reviewed and approved the final manuscript for submission.

## Data availability

All data generated or analyzed during this study are included in this published article.

## Funding

None. This research was conducted independently, driven solely by our academic interest and commitment to scientific inquiry. No funding was received to support this work, and none of the authors were compensated or received a salary specifically related to the development of this manuscript.

## Conflict of interest

The authors declare that they have no known competing financial interests or personal relationships that could have appeared to influence the work reported in this article.
