# Supplementary material for: Probiotics and Synbiotics Supplementation Reduce Inflammatory Cytokines in Individuals with Prediabetes and Type 2 Diabetes Mellitus: Findings from a Systematic Review Meta-analysis
Source: Adv Nutr. 2025 Sep 27;16(11):100526. doi: 10.1016/j.advnut.2025.100526 (PMC12590280; doi:10.1016/j.advnut.2025.100526)
Supplement: Multimedia component 1 [file mmc1.docx]

**Supplementary Materials**

**Supplementary Table 1.** Search Strategy

|  | **Query and search Keywords** |
| --- | --- |
| **Intervention** | “probiotics” OR “probiotic” OR “synbiotic” OR “synbiotics” OR “pro-biotic*” OR “syn-biotic*” OR “Lactobacillus” OR “bifidobacteria” OR “Bifidobacterium” |
| **Variables** | “Inflam*” OR “Inflammatory” OR “Inflammation” OR “Cytokines” OR “c-reactive protein” OR “CRP” OR “interleukin-6” OR “IL-6” OR “tumor necrosis factor” OR “tumor necrosis factor-alpha” OR “TNF” OR “TNF-α” |
| **Population** | “diabetes” OR “diabetic” OR “pre-diabetes” OR “pre-diabetic” OR “prediabetes” OR “prediabetic” OR “hyperglycemia” OR “hyperglycemic” |
| **Study design** | “randomized clinical trial” OR “randomized controlled trial” OR “RCT” OR “clinical trial” OR “trial” OR “controlled trial” OR “randomized” OR “random*” OR “intervention” OR “placebo” |

**Supplementary Table 2.** Search-line in databases (PubMed, ISI Web of Science, Scopus) (Title & Abstract) (Date: January 2025)

| **Databases** | **Search line** | **#** |
| --- | --- | --- |
| ***PubMed*** | ((("probiotics"[Title/Abstract] OR "probiotic"[Title/Abstract] OR "synbiotic"[Title/Abstract] OR "synbiotics"[Title/Abstract] OR "pro-biotic*"[Title/Abstract] OR "syn-biotic*"[Title/Abstract] OR "Lactobacillus"[Title/Abstract] OR "bifidobacteria"[Title/Abstract] OR "Bifidobacterium"[Title/Abstract]) AND ("Inflam*"[Title/Abstract] OR "Inflammatory"[Title/Abstract] OR "Inflammation"[Title/Abstract] OR "Cytokines"[Title/Abstract] OR "c-reactive protein"[Title/Abstract] OR "CRP"[Title/Abstract] OR "interleukin-6"[Title/Abstract] OR "IL-6"[Title/Abstract] OR "tumor necrosis factor"[Title/Abstract] OR "tumor necrosis factor-alpha"[Title/Abstract] OR "TNF"[Title/Abstract] OR "TNF-α"[Title/Abstract])) AND ("diabetes"[Title/Abstract] OR "diabetic"[Title/Abstract] OR "pre-diabetes"[Title/Abstract] OR "pre-diabetic"[Title/Abstract] OR "prediabetes"[Title/Abstract] OR "prediabetic"[Title/Abstract] OR "hyperglycemia"[Title/Abstract] OR "hyperglycemic"[Title/Abstract])) AND ("randomized clinical trial"[Title/Abstract] OR "randomized controlled trial"[Title/Abstract] OR "RCT"[Title/Abstract] OR "clinical trial"[Title/Abstract] OR "trial"[Title/Abstract] OR "controlled trial"[Title/Abstract] OR "randomized"[Title/Abstract] OR "random*"[Title/Abstract] OR "intervention"[Title/Abstract] OR "placebo"[Title/Abstract]) | 327 |
| ***ISI WoS*** | Results for “probiotics” OR “probiotic” OR “synbiotic” OR “synbiotics” OR “pro-biotic*” OR “syn-biotic*” OR “Lactobacillus” OR “bifidobacteria” OR “Bifidobacterium” (Topic) AND “Inflam*” OR “Inflammatory” OR “Inflammation” OR “Cytokines” OR “c-reactive protein” OR “CRP” OR “interleukin-6” OR “IL-6” OR “tumor necrosis factor” OR “tumor necrosis factor-alpha” OR “TNF” OR “TNF-α” (Topic) AND “diabetes” OR “diabetic” OR “pre-diabetes” OR “pre-diabetic” OR “prediabetes” OR “prediabetic” OR “hyperglycemia” OR “hyperglycemic” (Topic) AND “randomized clinical trial” OR “randomized controlled trial” OR “RCT” OR “clinical trial” OR “trial” OR “controlled trial” OR “randomized” OR “random*” OR “intervention” OR “placebo” (Topic) | 516 |
| ***Scopus*** | ( TITLE-ABS-KEY ( "probiotics" OR "probiotic" OR "synbiotic" OR "synbiotics" OR "pro-biotic*" OR "syn-biotic*" OR "Lactobacillus" OR "bifidobacteria" OR "Bifidobacterium" ) AND TITLE-ABS-KEY ( "Inflam*" OR "Inflammatory" OR "Inflammation" OR "Cytokines" OR "c-reactive protein" OR "CRP" OR "interleukin-6" OR "IL-6" OR "tumor necrosis factor" OR "tumor necrosis factor-alpha" OR "TNF" OR "TNF-α" ) AND TITLE-ABS-KEY ( "diabetes" OR "diabetic" OR "pre-diabetes" OR "pre-diabetic" OR "prediabetes" OR "prediabetic" OR "hyperglycemia" OR "hyperglycemic" ) AND TITLE-ABS-KEY ( "randomized clinical trial" OR "randomized controlled trial" OR "RCT" OR "clinical trial" OR "trial" OR "controlled trial" OR "randomized" OR "random*" OR "intervention" OR "placebo" ) ) | 1227 |
| ***All*** | PubMed (n=327), ISI Web of Science (n=516), Scopus (n=1227) | 2070 |

**Supplementary Table 3**. Meta-regression and Dose-response analysis

| **Variables** | **Regression** | | **Dose-response** | |
| --- | --- | --- | --- | --- |
|  | **Duration (week)** | | **Duration (week)** | |
|  | **Coefficient** | **p-value** | **Coefficient** | **p-value** |
| ***CRP*** | -0.26 | 0.772 | -0.58 | 0.540 |
| ***IL6*** | -1.38 | 0.599 | -1.31 | 0.168 |
| ***TNF-α*** | 0.09 | 0.913 | 1.22 | 0.608 |

**Supplementary Table 4.** Publication bias and Sensitivities analysis

| **Outcomes** | **Egger** | **Begg** | **Sensitivities** |
| --- | --- | --- | --- |
| ***CRP*** | 0.185 | 0.820 | None |
| ***IL6*** | 0.926 | 0.640 | Tay et al. 2020 (WMD: -0.35, 95%CI: -0.86, 0.15) |
| ***TNF-α*** | 0.443 | 0.754 | Hsieh et al. 2018 (WMD: -1.06, 95%CI: -2.22, 0.09)  Sabico et al. 2019 (WMD: -1.01, 95%CI: -2.29, 0.25)  Toejing et al. 2021 (WMD: -0.64, 95%CI: -1.43, 0.13)  Wei et al. 2022 (WMD: -0.72, 95%CI: -1.53, 0.09)  Savytska et al. 2023 (WMD: -0.81, 95%CI: -1.68, 0.04) |
